# Unemployment at municipality level is associated with an increased risk of small for gestational age births – a multilevel analysis of all singleton births during 2005–2010 in Finland

**DOI:** 10.1186/s12939-014-0095-1

**Published:** 2014-10-18

**Authors:** Sari Räisänen, Michael R Kramer, Mika Gissler, Juho Saari, Seppo Heinonen

**Affiliations:** Department of Epidemiology, Rollins School of Public Health, Emory University, 1518 Clifton Road NE, Atlanta, GA USA; Department of Obstetrics and Gynaecology, Kuopio University Hospital, P.O. Box 100, Kuopio, FI-70029 Kys Finland; National Institute for Health and Welfare (THL), P.O. Box 30, Paciuksenkatu 21, Helsinki, FI-00271 Finland; Nordic School of Public Health, Gothenburg, Sweden; Kuopio Welfare Research Centre (KWRC) and Department of Social Sciences, University of Eastern Finland, P.O. Box 1627, Kuopio, 70211 Finland; School of Medicine, University of Eastern Finland, P.O. Box 1627, Kuopio, FI-70211 Finland

**Keywords:** Childbirth, Multilevel analysis, Outcome, Preterm birth, Registries, Socioeconomic status, Small for gestational age

## Abstract

**Introduction:**

Neighbourhood level deprivation has been shown to influence adverse perinatal outcomes independent of individual level socioeconomic status (SES) in countries with high income inequality, such as the United States. The present study evaluates whether municipality level deprivation defined based on education (proportion of inhabitants with university level education), income (mean income per capita) and unemployment were associated with the prevalence of preterm birth (<37 weeks) and small for gestational age (SGA, birth weight <2 standard deviations) after adjustment for individual level socio-demographics (age, parity, prior preterm births, smoking during pregnancy and SES defined based on maternal occupation at birth) in Finland.

**Methods:**

The study design was cross-sectional. The data gathered from the Medical Birth Register included all singleton births (n = 345,952) in 2005–2010. We fitted Generalized Estimating Equations (GEE) models to account for correlation of preterm birth and SGA clustering within municipality.

**Results:**

Of all the women with singleton pregnancies, 4.5% (*n* = 15,615) gave birth preterm and 3.8% (*n* = 13,111) of their newborns were classified as SGA. Individual level SES and smoking were important risk factors for each outcome in adjusted models. Controlling for individual level factors, women living in intermediate and high unemployment class municipalities were 6.0% (adjusted odds ratio (aOR) = 1.06; 95% confidence interval (CI) 1.01-1.12) and 13.0% (aOR = 1.13; 95% CI 1.06-1.20), respectively, more likely to give birth to an SGA newborn than women living in low unemployment class municipalities.

**Conclusions:**

After adjustment for individual level socio-demographics, the prevalence of SGA was around 6-13% higher in municipalities with an intermediate or high unemployment rate than municipalities with the lowest unemployment rate. The results suggested that the unemployment rate has an important public health effect with clinical implications since SGA is associated with a higher risk of adverse long-term health outcomes.

## Introduction

Social health inequalities have been well demonstrated by numerous, previous epidemiological studies [1]. It has been suggested that after adjustment for individual level socioeconomic status (SES), neighbourhood level SES is independently associated with an increased burden of morbidity and mortality, including cardiovascular diseases (as reviewed by Chaix [2]) and all-cause mortality (as reviewed by Meijer et al. [3]). Several previous studies have also demonstrated an association between neighbourhood level SES and adverse perinatal outcomes, suggesting effects on the health of the next generation [4]. A systematic review and meta-analysis by Metcalfe et al. demonstrated an association between neighbourhood income and low birth weight (LBW, <2500 g) after adjustment for individual level socio-demographics [4]. However, most of the studies included in the review were from the United States, and thus their results might not be generalizable to other industrialised countries with different economic characteristics, including Nordic welfare states [4]. We have identified only three previous studies in European populations, which test for the association between neighbourhood level deprivation and adverse perinatal outcomes while controlling for individual level SES and behaviours. Consideration of these variables is important because each is strongly correlated with neighbourhood deprivation and adverse perinatal outcomes [5]. Two previous studies with small random samples from the Netherlands reported inconsistent results; neighbourhood level deprivation was an independent risk factor for giving birth to a small for gestational age (SGA) newborn (birth weight below 10^th^ percentile of birth weight standard), [6] and a greater prevalence of adverse perinatal outcomes in deprived neighbourhoods was explained by individual level characteristics [7]. Furthermore, a previous large population based cohort study from Sweden (*n* = 330,096) suggested that neighbourhood level deprivation had only a minor influence on the mean birth weight [8].

Several mechanisms or pathways for explaining how neighbourhood level circumstances might influence individual level health have been proposed. It has been suggested that neighbourhood level SES (measured based on income and unemployment) and social-capital (such as social networks, social participation and social trust) influence environmental exposures, such as stress, material resources, and quality and availability of healthcare and other services [9-11].

In the present study, we reviewed all singleton births contained in the Finnish Medical Birth Register (MBR) during 2005–2010. The research aim was to evaluate whether municipality-level deprivation defined based on education (proportion of inhabitants with university level education), income (mean income per capita) and unemployment influences the geographic variation of preterm birth (<37 weeks) and SGA (birth weight below 2 standard deviations) after adjustment for individual level socio-demographics (age, parity, prior preterm births, smoking during pregnancy, and SES defined based on maternal occupation at birth) in Finland, a Nordic welfare state. Welfare states promote social cohesion and reduce inequality by normalising access to social and health care services and education, which are mostly publicly produced and funded by taxes.

## Methods

### Data

Data from the Finnish Medical Birth Register (MBR) were acquired from the National Institute for Health and Welfare (THL) (http://www.thl.fi/en/web/thlfi-en/statistics/information-on-statistics/register-descriptions/newborns). The MBR includes information collected during the first seven postnatal days on socio-demographics, maternal reproductive history, pregnancy and delivery characteristics and maternal and newborn diagnoses defined and coded based on the International Classification of Diseases (ICD-10). Information on previous preterm births (yes or no) was gathered from the MBR based on the reported ICD-10 codes.

### Population

The study population included all women with singleton births (*N* = 345,952) during 2005–2010 in Finland but excluded births with missing information for birth weight (*n* = 692), gestational age (*n* = 1,162), and parity (*n* = 368) as well as children with undetermined fetal sex (*n* = 17). In total, 1,162 and 1,377 cases were excluded from the analysis of preterm birth and SGA, respectively. Multiple births (*n* = 10,653) were excluded due to their higher risk of adverse perinatal outcomes.

### Variables and definitions

#### Individual level information

Preterm birth was defined as gestational weeks lower than 37. Newborns were classified as SGA if the birth weight was at least 2 standard deviations (SDs) below the sex- and gestational age-specific reference mean according to the Finnish population-based reference for birth size [12]. MBR records the best estimate for gestational age at birth, which is practically always based on first- or second-trimester ultrasonography measurements. Maternal age was considered a categorical variable and classified into four groups: less than 20, 20–29, 30–39 and 40 years or more. Based on prior childbirths, parity was classified as either nulliparous (no prior childbirths) or multiparous (one or more prior childbirths). Based on self-reported smoking habits during pregnancy (reviewed during antenatal visits during pregnancy), women were divided into three classes: non-smoking, stopped smoking during the first pregnancy trimester, continued smoking after the first pregnancy trimester. A previous study has reported validity of information on smoking in MBR to be high [13]. Women with missing information on smoking (*n* = 9,237, 2.7%) were included in the analyses as a separate group. Women were also classified based on their occupation at birth into five socioeconomic status (SES) groups according to Finland's National Classification of Occupations, [14] which follows international recommendations. SES was classified as either upper white-collar workers (the highest SES), such as physicians and lawyers; lower white-collar workers, such as nurses and secretaries; blue-collar workers (the lowest SES), such as cooks and cleaners; others; and missing information, as categorized and published elsewhere [15]. ‘Others’ included all unclassifiable categories, such as students, entrepreneurs, retired, unemployed and housewives. The category with missing information on SES comprised 20.5% (*n* = 71,072) of the total included cases. Information on the municipalities where the parturients lived was based on information at birth. Annual preterm birth and SGA rates were examined to evaluate secular trends.

### Residential area level information

The municipalities corresponded to a local level of administration and served as the fundamental, self-governing administrative unit that defined the residential areas. During the study period, the number of municipalities reduced from 432 in 2005 to 342 in 2010 due to small population municipalities being combined. In 2013, Finland comprised 320 municipalities that were considered as discrete residential areas in the present study. The municipalities differed by area and population size; the population size varied from 100 to 600,000. To account for this variation a variable was created by dividing municipalities into tertiles by population size: first tertile between 100 and 21,000 inhabitants; second tertile more than 21,000 and less than 106,000 inhabitants; and third tertile more than 106,000 and up to 600,000 inhabitants. Of all the inhabitants, 78% lived in an urbanized area according to the proportion of people living in urban settlements and the population of the largest urban settlement as defined by Statistics Finland http://www.stat.fi/meta/kas/til_kuntaryhmit_en.html. Municipalities are responsible for organizing services such as healthcare, social services and infrastructure as set by the laws in Finland with support from the state. (http://www.localfinland.fi/en/authorities/local-self-government/Documents/Finnish%20Local%20Government%20Act.pdf).

Three municipality level deprivation indices were used; income (annual mean income per capita), education (proportion of inhabitants with university level education) and unemployment (residential area unemployment rate). Each index was categorized into tertiles of the number of parturients. Tertiles were labelled from low to high for education, income and unemployment based on the following cut-off points: *Income;* low (up to 25,000 euro, 227 municipalities), intermediate (more than 25,000 but less than 28,500 euro, 57 municipalities) and high (28,500 euro or more, 36 municipalities). *Education;* low (up to 25.4%, 266 municipalities), intermediate (more than 25.4% but less than 32.3%, 38 municipalities) and high (32.3% or more, 16 municipalities). *Unemployment*; high (up to 12.0%, 106 municipalities), intermediate (at least 8.0% but less than 12.0%, 128 municipalities) and low (7.9% or less, 86 municipalities). We also acquired information on the population size of each municipality. Information on the municipalities was gathered from Finnish Statistics (http://www.stat.fi/) for 2011.

### Statistical analyses

Differences between the groups (preterm births vs. term births [37 weeks or more] and SGA newborns vs. not SGA newborns) were evaluated by chi square test for dichotomous and categorical variables and by Student’s *t*-test for continuous variables. To account for the correlation of observations clustered within each municipality, we fitted Generalized Estimating Equations (GEE) models. A base model was adjusted by year of birth only (Model 1), and then additional covariates were added in subsequent models: adjustment for individual level covariates (Model 2), adjustment for municipality level covariates (Model 3), adjustment for all individual level covariates except smoking and municipality level covariates (Model 4), and finally adjustment for all individual and municipality level covariates (Model 5). The contribution of individual level smoking on deprivation was measured based on (OR Model 4 – OR Model 5)/(OR Model 4 – 1). All individual level covariates except year of birth and maternal age were used as categorical variables. An association between maternal age and outcomes was not linear, but it was used as a continuous variable since results did not change after using non-linear fits. Municipality level deprivation indices were used as categorical variables and the log of population was used as a continuous variable in the GEE models. The log of population count was entered in the models because the municipality population size distribution was skewed.

Multicollinearity between municipality level variables could have led to statistical problems. Therefore, we performed Spearman correlation tests, which gave *r* values between 0.24 and 0.64. To assess the sensitivity of the results to missing SES information, we performed multiple imputation analysis. Differences were deemed to be significant if the *p*-value was less than 0.05. The data were analyzed using SPSS for Windows 19.0, Chicago, IL.

### Ethics approval

Study plan and permission to use the confidential register data in this study was granted on 16^th^ February, 2012 by the National Institute for Health and Welfare (THL) in Finland. (Reference number 1749/5.05.00/2011).

## Results

In total, 4.5% (*n* = 15,615) of all the included women with singleton pregnancies gave birth preterm (<37 weeks) and 3.8% (*n* = 13,111) of all the newborns were classified as SGA (<2 SD below mean birth weight). Women who gave birth preterm or to an SGA newborn were more likely to be young (less than 20 years), of advanced age (40 years or more), nulliparous, continued smoking after the first trimester and in the lowest SES group (blue white collar worker) than women who gave birth in term or to a non-SGA newborn (Table 1). The prevalence of preterm birth varied significantly by tertile of municipality education, income and unemployment classes; giving birth preterm was less frequent in the wealthiest and most prosperous municipalities. The prevalence of SGA varied significantly only by municipality unemployment classes; giving birth to a SGA newborn was more frequent in the most deprived municipalities defined based on unemployment.

**Table 1:** **Socio-demographic characteristics of preterm, term, small for gestational age (SGA) inewborns and not SGA newborns among women with singleton births during 2005–2010 in Finland**

**Characteristic**	**Preterm birth (<37 weeks)**	**Term (≥37 weeks)**	* ***p*** **value**	**SGA (<2 SD)**	**Not SGA**	* ***p*** **value**
Individual level information						
*n* (%)	15,615 (4.5)	330,337		13,111 (3.8)	332,626	
Mean maternal age, years (SD)	29.8 (5.7)	29.5 (5.4)	≤0.001	29.1 (5.9)	29.5 (5.4)	≤0.001
*Maternal age, years %*			≤0.001			≤0.001
<20	5.7	94.3		6.6	93.4	
20-29	4.3	95.7		3.9	96.1	
30-39	4.5	95.5		3.5	96.5	
40 or more	6.1	93.9		4.7	95.3	
*Prior preterm birth*			≤0.001			0.016
Yes	14.5	85.5		4.2	95.8	
No				3.8	96.2	
*Parity %*						≤0.001
Nulliparous women	5.3	94.7	≤0.001	6.2	93.8	
Multiparous women	3.9	96.1		2.1	97.9	
*Smoking status*						≤0.001
Non-smoking	4.3	95.7		3.3	96.7	
Quit smoking during 1^st^ trimester	4.5	95.5		3.7	96.3	
Smoking after 1^st^ trimester	5.6	94.4		7.5	92.5	
Missing information	8.2	91.8		3.8	96.2	
*Socioeconomic status %*			≤0.001			≤0.001
Upper white collar workers	4.0	96.0		3.2	96.8	
Lower white collar workers	4.5	95.5		3.6	96.4	
Blue collar workers	4.7	95.3		4.1	95.9	
Others^a^	4.3	95.7		3.8	96.2	
Missing information	4.9	95.1		4.1	95.9	
*Year*			0.531			0.017
2005	4.5	95.5		3.7	96.3	
2006	4.7	95.3		3.6	96.4	
2007	4.5	95.5		4.0	96.0	
2008	4.5	95.5		3.7	96.3	
2009	4.4	95.6		3.9	96.1	
2010	4.5	95.5		3.8	96.2	
Municipality level information						
*Education deprivation class*			0.003			0.116
High (least deprived)	4.3	95.7		3.8	96.2	
Intermediate	4.6	95.4		3.9	96.1	
Low (most deprived)	4.6	95.4		3.7	96.3	
*Income deprivation class*			≤0.001			0.574
High (least deprived)	4.3	95.7		3.8	96.2	
Intermediate	4.6	95.4		3.7	96.3	
Low (most deprived)	4.6	95.4		3.8	96.2	
*Unemployment deprivation class*			≤0.001			≤0.001
Low (least deprived)	4.3	95.7		3.7	96.3	
Intermediate	4.6	95.4		3.7	96.3	
High (most deprived)	4.7	95.3		4.0	96.0	
*Tertiles of municipality by population size*			≤0.001			0.04
First tertile (100–21,000)	3.5	96.5		4.5	95.5	
Second tertile (21,000-106,000)	3.9	96.1		4.6	95.4	
Third tertile (106,000-600,000)	3.9	96.1		4.4	95.6	

^a^Others comprised entrepreneurs, students, retired women, unemployed women, housewives and all unclassifiable cases, *Student’s *t* or chi square test, SD = standard deviation.

Table 2 presents results of the GEE models of preterm birth. Model 1 (adjusted for year of birth) showed that the prevalence of preterm birth did not vary significantly by year of birth. Model 2 (adjusted for all individual level covariates) showed that a higher prevalence of preterm birth was associated with maternal age, nulliparity, prior preterm birth, continuing smoking after the first trimester, and low SES. Model 3 showed the prevalence of preterm birth after adjustment for municipality level covariates. Model 5 (adjusted for all individual and municipality level covariates) showed that the prevalence of preterm birth varied significantly only between high and intermediate deprivation indices determined based on education. The prevalence of preterm birth was 6.0% (aOR 1.06, 95% CI 1.01 to1.11) higher in intermediate education class municipalities than high education class municipalities. Differences between classes of municipalities, determined based on income and unemployment, were not significant.

**Table 2:** **Parameter estimates for preterm birth (n = 15,593) associated with individual and area level characteristics among all singleton births (n = 330,688) during 2005–2010 in Finland**

**Characteristics**	**Model 1 adjusted for year of birth**	**Model 2 adjusted for individual level confounders**	**Model 3 adjusted for municipality level confounders**	**Model 4 adjusted for model 2 (except smoking) and Model 3**	**Model 5 adjusted for model 2 and model 3**
	OR (95% CI)	aOR (95% CI)	aOR (95% CI)	aOR (95% CI)	aOR (95% CI)
Year of birth	0.99 (0.99-1.00)	0.99 (0.98-1.00)		0.99 (0.98-1.00)	0.99 (0.98-1.00)
Maternal age, years (per one year increase)		1.02 (1.02-1.03)		1.02 (1.02-1.02)	1.02 (1.02-1.03)
*Parity*					
Nulliparous women		1		1	1
Multiparous women		0.52 (0.50-0.54)		0.52 (0.50-0.54)	0.51 (0.49-0.53)
Prior preterm birth		5.28 (5.00-5.57)		5.30 (5.03-5.59)	5.27 (4.99-5.56)
*Smoking status*					
Non-smoking		1			1
Quit smoking during 1^st^ trimester		1.03 (0.95-1.12)			1.03 (0.95-1.11)
Smoking after 1^st^ trimester		1.36 (1.29-1.43)			1.35 (1.29-1.42)
Missing information		2.05 (1.89-2.22)			2.02 (1.86-2.19)
*Socioeconomic status*					
Upper white collar workers		1		1	1
Lower white collar workers		1.15 (1.07-1.23)		1.15 (1.07-1.23)	1.13 (1.06-1.21)
Blue collar workers		1.19 (1.11-1.29)		1.23 (1.14-1.33)	1.18 (1.09-1.27)
Others^a^		1.11 (1.03-1.19)		1.12 (1.05-1.20)	1.10 (1.03-1.18)
Missing information		1.27 (1.14-1.32)		1.27 (1.18-1.36)	1.24 (1.15-1.33)
*Municipality derivation class* ^b^					
*Education*					
High (least deprived)			1	1	1
Intermediate			1.05 (1.00-1.10)	1.06 (1.01-1.11)	1.06 (1.01-1.11)
Low (most deprived)			1.03 (0.97-1.10)	1.03 (0.97-1.10)	1.03 (0.96-1.10)
*Income*					
High (least deprived)			1	1	1
Intermediate			1.03 (0.98-1.09)	1.05 (0.99-1.11)	1.05 (0.99-1.11)
Low (most deprived)			1.05 (0.98-1.12)	1.07 (1.00-1.14)	1.06 (0.99-1.14)
*Unemployment*					
Low (least deprived)			1	1	1
Intermediate			1.02 (0.98-1.07)	1.03 (0.98-1.08)	1.02 (0.98-1.08)
High (most deprived)			1.04 (0.99-1.10)	1.05 (1.00-1.11)	1.05 (0.99-1.11)
Log (population)			1.01 (0.99-1.02)	1.00 (0.98-1.01)	1.00 (0.98-1.02)

^a^Others comprised entrepreneurs, students, retired women, unemployed women, housewives and all unclassifiable cases.

^b^Municipality level education (percentage of inhabitants with university level education), income (annual mean income per inhabitant) and unemployment (percentage of unemployed inhabitants) were stratified based on tertiles of number of parturients into three classes equal in population size (number of parturients).

OR=adjusted odds ratio, CI=confidence interval.

Table 3 presents results of the GEE models of SGA newborns. Model 1 (adjusted for year of birth) showed that the prevalence of SGA newborns increased by 1% per year during 2005–2010. Model 2 (adjusted for all individual level confounders) showed that a higher prevalence of a SGA newborn was associated with maternal age, nulliparity, prior preterm birth, continuing smoking after first trimester, and low SES. Model 3 showed the prevalence of a SGA newborn after adjustment for municipality level confounders. Model 5 (adjusted for all individual and municipality level confounders) showed that the prevalence of a SGA newborn varied significantly by municipality level deprivation determined based on unemployment. The prevalence of a SGA newborn was 6.0% (aOR 1.06, 95% CI 1.01 to1.12) higher in intermediate unemployment class municipalities and 13.0% (aOR 1.13, 95% CI 1.06 to1.20) higher in high unemployment class municipalities than low unemployment class municipalities. Differences between tertiles of municipalities, determined based on education and income, were not significant. It appeared that individual level smoking mediated area deprivation and explained up to 57.1% and 13.3% [(OR Model 4 – OR Model 5)/(OR Model 4 – 1)] of SGA associated with municipality deprivation determined based on education and unemployment, respectively.

**Table 3:** **Parameter estimates for small for gestational age (SGA) newborn (n = 13,146) associated with individual and area level characteristics among all singleton births (n = 346,281) during 2005–2010 in Finland**

**Characteristic**	**Model 1 adjusted for year of birth**	**Model 2 adjusted for individual level confounders**	**Model 3 adjusted for municipality level confounders**	**Model 4 adjusted for model 2 (except smoking) and Model 3**	**Model 5 adjusted for model 2 and model 3**
	OR (95% CI)	aOR (95% CI)	aOR (95% CI)	aOR (95% CI)	aOR (95% CI)
Year of birth	1.01 (1.00-1.02)	1.01 (1.00-1.02)		1.01 (1.00-1.02)	1.01 (1.00-1.02)
Maternal age, years (per one year increase)		1.03 (1.03-1.04)		1.02 (1.02-1.03	1.03 (1.03-1.04)
*Parity*					
Nulliparous women		1		1	1
Multiparous women		0.27 (0.26-0.28)		0.27 (0.26-0.28)	0.27 (0.25-0.28)
Prior preterm birth		2.14 (1.96-2.34)		2.20 (2.01-2.40)	2.14 (1.96-2.34)
*Smoking status*					
Non-smoking		1			1
Quit smoking during 1^st^ trimester		0.96 (0.88-1.05)			0.96 (0.88-1.05)
Smoking after 1^st^ trimester		2.47 (2.35-2.59)			2.45 (2.34-2.57)
Missing information		1.34 (1.19-1.49)			1.32 (1.18-1.48)
*Socioeconomic status*					
Upper white collar workers		1		1	1
Lower white collar workers		1.179 (1.09-1.26)		1.22 (1.13-1.31)	1.16 (1.08-1.25)
Blue collar workers		1.21 (1.11-1.32)		1.39 (1.28-1.51)	1.20 (1.10-1.30)
Others^a^		1.17 (1.09-1.27)		1.23 (1.14-1.32)	1.17 (1.08-1.26)
Missing information		1.19 (1.10-1.29)		1.29 (1.20-1.40)	1.20 (1.111.29)
*Municipality derivation class* ^b^					
*Education*					
High (least deprived)			1	1	1
Intermediate			1.09 (1.03-1.14)	1.07 (1.01-1.13)	1.03 (0.98-1.09)
Low (most deprived)			1.12 (1.04-1.20)	1.11 (1.03-1.19)	1.05 (0.97-1.13)
*Income*					
High (least deprived)			1	1	1
Intermediate			0.94 (0.88-0.99)	0.93 (0.87-0.99)	0.94 (0.89-1.00)
Low (most deprived)			0.99 (0.92-1.07)	0.99 (0.92-1.07)	1.01 (0.94-1.09)
*Unemployment*					
Low (least deprived)			1	1	1
Intermediate			1.03 (0.98-1.09)	1.07 (1.01-1.12)	1.06 (1.01-1.12)
High (most deprived)			1.12 (1.06-1.19)	1.15 (1.08-1.22)	1.13 (1.06-1.20)
Log (population)			1.06 (1.04-1.08)	1.01 (1.00-1.03)	1.01 (0.99-1.03)

^a^Others comprised entrepreneurs, students, retired women, unemployed women, housewives and all unclassifiable cases.

^b^Municipality level education (percentage of inhabitants with university level education), income (annual mean income per inhabitant) and unemployment (percentage of unemployed inhabitants) were stratified based on tertiles of number of parturients into three classes equal in population size (number of parturients).

OR=adjusted odds ratio, CI=confidence interval.

Multiple data imputations of missing information on SES did not change the results (Model 5 presented for both outcomes in Table 4).

**Table 4:** **Adjusted odds ratios (aOR) for preterm birth (n = 15,593), and small for gestational age (SGA) newborn (n = 13,146) associated with individual and area level characteristics (using multiple imputed data) among all singleton births (n = 330,688) during 2005–2010 in Finland**

**Characteristics**	**Preterm birth**	**SGA**
	**aOR (95% CI)**	**aOR (95% CI)**
Year of birth	1.00 (0.99-1.01)	1.01 (1.00-1.02)
Maternal age, years (per one year increase)	1.02 (1.02-1.03)	1.04 (1.03-1.04)
*Parity*		
Nulliparous women	1	1
Multiparous women	0.52 (0.50-0.54)	0.26 (0.25-0.28)
Prior preterm birth	5.27 (4.99-5.55)	2.14 (1.96-2.34)
*Smoking status*		
Non-smoking	1	1
Quit smoking during 1^st^ trimester	1.03 (0.95-1.11)	0.95 (0.87-1.03)
Smoking after 1^st^ trimester	1.37 (1.30-1.43)	2.45 (2.33-2.56)
*Socioeconomic status*		
Upper white collar workers	1	1
Lower white collar workers	1.13 (1.06-1.19)	1.18 (1.11-1.27)
Blue collar workers	1.20 (1.12-1.28)	1.21 (1.13-1.31)
Others^a^	1.09 (1.02-1.16)	1.18 (1.10-1.27)
*Municipality derivation class* ^b^		
*Education*		
High (least deprived)	1	1
Intermediate	1.04 (0.99-1.09)	1.02 (0.97-1.08)
Low (most deprived)	1.01 (0.95-1.08)	1.04 (0.97-1.12)
*Income*		
High (least deprived)	1	1
Intermediate	1.04 (0.99-1.10)	0.94 (0.88-1.00)
Low (most deprived)	1.06 (0.99-1.14)	1.00 (0.93-1.08)
*Unemployment*		
Low (least deprived)	1	1
Intermediate	1.03 (0.98-1.08)	1.07 (1.01-1.12)
High (most deprived)	1.04 (0.98-1.08)	1.13 (1.06-1.20)
Log (population)	1.00 (0.98-1.01)	1.01 (0.99-1.03)

^a^Others comprised entrepreneurs, students, retired women, unemployed women, housewives and all unclassifiable cases.

^b^Municipality level education (percentage of inhabitants with university level education), income (annual mean income per inhabitant) and unemployment (percentage of unemployed inhabitants) were stratified based on tertiles of number of parturients into three classes equal in population size (number of parturients).

CI = confidence interval.

## Discussion

We reviewed all singleton births (*n* = 345,952) during 2005–2010 in Finland to measure whether municipality level education, income and employment were associated with a burden of preterm birth and giving birth to a SGA newborn after adjustment for individual level socio-demographics. Our results suggested that the prevalence of preterm birth and SGA newborns was associated with individual level low SES and smoking during pregnancy, but having a SGA newborn was also independently associated with municipality level deprivation defined by the aggregate unemployment rate. Women living in municipalities with an intermediate or high unemployment rate were 6.0% and 13.0%, respectively, more likely to give birth to a SGA newborn than women living in municipalities with a low unemployment rate.

### Strengths and weaknesses

The most important strength of the present study was that it utilized information on all recent singleton births in Finland contained within the Finnish MBR with high data quality and coverage [16]. The data were sufficiently large and comprehensive to detect differences in the prevalence of preterm birth and SGA newborns between municipality level deprivation classes. A further strength was that we examined the influence of municipality based deprivation by using three municipality level variables (income, education and unemployment).

The most important limitation of the present study was that information on SES was missing in one out of five parturients. Due to its sensitive nature, an increasing number of women probably chose not to provide such information. To reduce the bias caused by missing information on SES, we performed multiple imputations, but the results were unchanged, suggesting that women with missing SES represented all SES categories. Another limitation concerning SES was that its definition was solely based on the maternal occupation at birth, and we did not have individual level information on the women’s education, income or spouse for reasons related to data protection and privacy. Among the Finnish population, however, education and income are usually related to occupation, which is therefore an appropriate indicator for studies on socioeconomic health disparity [17,18]. Furthermore, the women’s place of residence was based on information at birth and we did not know how long they had been living in the municipality.

### Meaning and implications of the study

The main finding of the present study was that area-based deprivation matters even in a Nordic welfare state, where access to healthcare and social welfare services, such as minimal income for safe housing and food, are available to all citizens and permanent residents. Based on our results, we cannot explain the association, but it might be speculated that living in areas of greater deprivation may result in higher levels of accumulated chronic stress from social disorder and poverty, which can have physiologic implications for pregnancy, e.g., affecting birth weight [9,19]. Municipalities might also vary with respect to social capital and contacts that in turn may be linked to social structures and the quality of social and health services. These connections allow individuals to tap into a broader network of support and information in times of need, which has shown to have a direct effect on adverse birth outcome [9] and health behaviour [10]. Furthermore, while access to healthcare services is guaranteed, the quality of healthcare services might vary by area and its socio-demographic and economic status.

In the present study, among the three measures of deprivation investigated, only the unemployment rate and education were significantly associated with adverse perinatal outcomes. However, the association between unemployment and the prevalence of SGA newborns appeared to be stronger and dose dependent compared to any association between education and preterm birth. It might be speculated that municipalities with higher than average unemployment may have exceptional circumstances rather than there just being some individuals that are out of work. High regional unemployment may mark a decline of local industries and the concomitant decline in area institutions. Further, high unemployment may result in outward migration of the youngest and healthiest and concentration of higher risk individuals. Our results are partially consistent with a previous study on a small randomly selected cohort from the Netherlands, which reported a higher incidence of SGA newborns (birth weight below the 10^th^ percentile of birth weight standard) but not preterm births among women who lived in neighbourhoods with the highest unemployment or higher proportion of inhabitants receiving social benefits or having low income after adjustment for individual level SES and smoking [6]. It has been suggested that area-based psycho-social risk factors play a more important role in low birth weight than in preterm birth [9].

Overall, it should be noted that an association between SGA and municipality level unemployment was modest. Unmeasured individual such as lifestyle and municipality level factors might have biased the results. Further, we were not able to adjust genetic factors using for example siblings as a proxy, since we were not able to link pregnancies of the same woman. A previous study showed that effect of maternal smoking on birth weight was smaller in sibling analysis than in conventional analysis [20]. One problem of multilevel causal hypotheses is that it is not easy to distinguish which variables might confound an association and which may in fact be mediating ones. In the present study, we observed that the relationship between high vs. low deprivation municipalities (determined based on education) and SGA was stronger without adjustment for smoking; the magnitude of association was reduced by approximately half when the adjustment for smoking was included. Smoking is a complex behavioural trait, but there is evidence that the propensity to smoke (and keep smoking) is affected not only by individual factors but also by the norms of the social network around an individual [21,22]. Therefore, places characterized by higher smoking prevalence may affect an individual’s decisions about starting or quitting smoking. Smoking is a known strong risk factor for SGA, and thus it is not surprising that the association is diminished when taken into account. However, it is hard to assess whether smoking is a consequence of area-based deprivation or just a confounder.

## Conclusions

Our results showed that independent of individual level SES and smoking, the municipality-level unemployment rate was associated with a greater prevalence of SGA newborns, which itself is associated with an increased risk of several chronic diseases and influences on life course [23]. While much research steers clear of contextual explanations for population health patterns out of concern for the ecological fallacy, the inverse, the atomistic fallacy, which assumes individuals are unaffected by their environment, may be equally suspect. In our multilevel study, we jointly considered both individual and ecological variables, finding relevance in each. As social animals, humans have evolved in communities where social norms and status structures have played a central role in selection and survival. Public health efforts focusing exclusively or mainly on individualistic variables and factors like smoking and SES may neglect other important explanations derived from national and local policy, social structures and cultural configurations.

A 13% difference in the SGA prevalence between the high and low unemployment class municipalities is a clinically important finding. Based on our previous study utilizing the same data for a longer time period (1987–2010) we know that the prevalence of SGA was 26% (95% CI 21-29%) higher in women with a prior caesarean section (CS) [24]. In clinical practise an increased risk of adverse perinatal outcomes associated with a prior CS is a well-known risk factor; but the 13% increased risk associated with municipality level high unemployment, being half of the risk related to prior CS, is not at all considered as a risk factor. While correlation does not necessarily imply causation, these results may indicate that a reduction of the unemployment rate through various activation measures and social investments in low-SES areas, especially those with high unemployment and large number of “dislocated” individuals, might be beneficial not only for economic reasons but also for public health by reducing poor perinatal outcomes and the morbidity of the next generations. Simultaneously, more systematic attention should be focused on various collective processes that may nudge social behavior towards healthier lifestyles.
